# Clinician-Scientists in-and-between Research and Practice: How Social Identity Shapes Brokerage

**DOI:** 10.1007/s11024-020-09420-7

**Published:** 2020-10-06

**Authors:** Esther de Groot, Yvette Baggen, Nienke Moolenaar, Diede Stevens, Jan van Tartwijk, Roger Damoiseaux, Manon Kluijtmans

**Affiliations:** 1 Department of General Practice, Julius Center for Health Sciences and Primary Care, University Medical Center Utrecht, Utrecht University, Heidelberglaan 100, 3584 CX Utrecht, The Netherlands; 2 Human Resources Department, NN-Group, The Hague, The Netherlands; 3 Department of Education, Utrecht University, Utrecht, The Netherlands; 4 Dutch Inspectorate of Education, Ministry of Education, Culture and Science, Utrecht, The Netherlands; 5 NSO-CNA Leiderschapsacademie, Amsterdam, The Netherlands; 6 Department of Education, Utrecht University, Utrecht, The Netherlands; 7 Center for Education, University Medical Center Utrecht, Utrecht University, Utrecht, The Netherlands

**Keywords:** Clinician scientists, Medical professions, Social identity, Brokerage types

## Abstract

Clinician-scientists (CSs) are vital in connecting the worlds of research and practice. Yet, there is little empirical insight into how CSs perceive and act upon their in-and-between position between these socio-culturally distinct worlds. To better understand and support CSs’ training and career development, this study aims to gain insight into CSs’ social identity and brokerage. The authors conducted semi-structured, in-depth interviews with 17, purposively sampled, CSs to elicit information on their social identity and brokerage. The CSs differ in how they perceive their social identity. Some CSs described their social identity strongly as either a research or clinical identity (dominant research or clinical identity). Other CSs described combined research and clinical identities, which might sometimes be compartmentalised, intersected or merged (non-dominant-identity). In the types of brokerage that they employ, all CSs act as representatives. CSs with a non-dominant identity mostly act as liaison and show considerable variability in their repertoire, including representative and gatekeeper. CSs with a dominant identity have less diversity in their brokerage types. Those with a dominant research identity typically act as a gatekeeper. Combining lenses of social identity theory and brokerage types helps understand CSs who have a dual position in-and-between the worlds of clinical practice and research. Professional development programs should explicitly address CSs’ professional identities and subsequent desired brokerage. Research and policy should aim to clarify and leverage the position of CSs in-and-between research and practice.

## Introduction

Our society is getting ever more specialised with the risk of different disciplinary fields being insufficiently interconnected to address big societal challenges. An area in which the weak connection is urgently felt and described is the gap between (bio)medical research and clinical practice. In an influential publication, Butler referred to this gap as “the valley of death” (Butler 2008) because few research findings ever reach clinical practice. Mending the gap is not easy because research and clinical practice are inherently different in nature, with large socio-cultural differences (Roberts et al. 2012; Rosenblum et al. 2016). Multiple boundaries, professional and institutional, exist between science and clinical practice, which has implications for the social identity of clinician-scientists who work across these boundaries (Rowland and Ng 2017). Clinician-scientists (CSs) are practising clinicians who, in addition, are engaged in scientific research (Rosenblum et al. 2016). By being a member of both fields, they are uniquely positioned to facilitate exchange between research and practice, and as such are deemed vital to the advancement of medical practice (Barry et al. 2019; Lemoine 2008; Vignola-Gagné 2014; Wilson-Kovacs and Hauskeller 2012). By combining practice and research, CSs may act as *brokers* between distinct professional worlds, for instance, by transferring the latest insights from research to clinical practice and ensuring the clinical relevance of research (Hendriks et al. 2019; Kluijtmans et al. 2017; Roberts et al. 2012; Yanos and Ziedonis 2006). While this brokerage may motivate and enrich CSs, it also comes next to the already demanding role to perform well in both clinical practice and research (Kluijtmans et al. 2017; Roberts et al. 2012). It is not hard to imagine the difficulties for CSs to perform in both worlds and on top of that broker between those worlds. We see an increasing volume of literature showing that their dual position is demanding and not well supported (Roberts et al. 2012; Rosenblum et al. 2016; Yanos and Ziedonis 2006). They often feel they are undervalued by having their output compared to full-time colleagues, whilst their efforts in the second field are not being taken into account (Croft et al. 2015). Despite initial interest and motivation, many early-career CSs choose to focus on research or clinical activity only (Edelman and LaMarco 2012). The difficulties of maintaining a dual career may explain why, despite their recognised importance, their numbers have for several decades been declining (National Institutes of Health 2014; Schafer 2010). Three lines of action have been proposed to remediate CS shortages: a better understanding of the nature of the CS role, improve reward system to account for dual-position and strengthen recognition of brokerage (Weggemans et al. 2019). So far, only a limited number of studies have concentrated on the first recommendation: understanding the nature of CS brokerage. This study aims to add to this strand of research. It investigates how CSs perceive themselves and how that shapes their brokerage to educate better, acknowledge and support CSs in their enactment of connectors between research and practice.

### CSs: A Social Identity Perspective

Kluijtmans and colleagues (2017) conducted an explorative study on the identity development of early-career CSs in nursing and physiotherapy. Their results suggest that early-career CSs developed dual identities as clinicians and scientists to operate in both fields and brokerage of evidence- and practice-based knowledge. Typically, they also developed a third meta-identity as a “broker”, describing themselves as “bridgers” between worlds, allowing them to adapt their behaviour to situational demands and deal with potential tensions between both fields.

Social identity complexity theory (Roccas and Brewer 2002) could help to understand identities and identity development of CSs better. Roccas and Brewer (2002) explain that people are a member of different social groups and, as a result, have to reconcile multiple social identities—which is precisely the case in the working life of CSs. A person’s identity determines what social group is recognised as *in-group* and, accordingly, people are perceived as members of the *in-group* or *out-group*. In the case of CSs, they may identify clinicians and scientists either as in-group or out-group. As clinical practice and science differ substantially, identifying both clinicians and scientists as in-group members is not self-evident. Roccas and Brewer (2002) argue that it is mainly in such cases—when social groups differ on several dimensions—essential to understanding multiple social identity development because this affects how a person encounters distinct professional worlds and relationships.

**Fig. 1:**
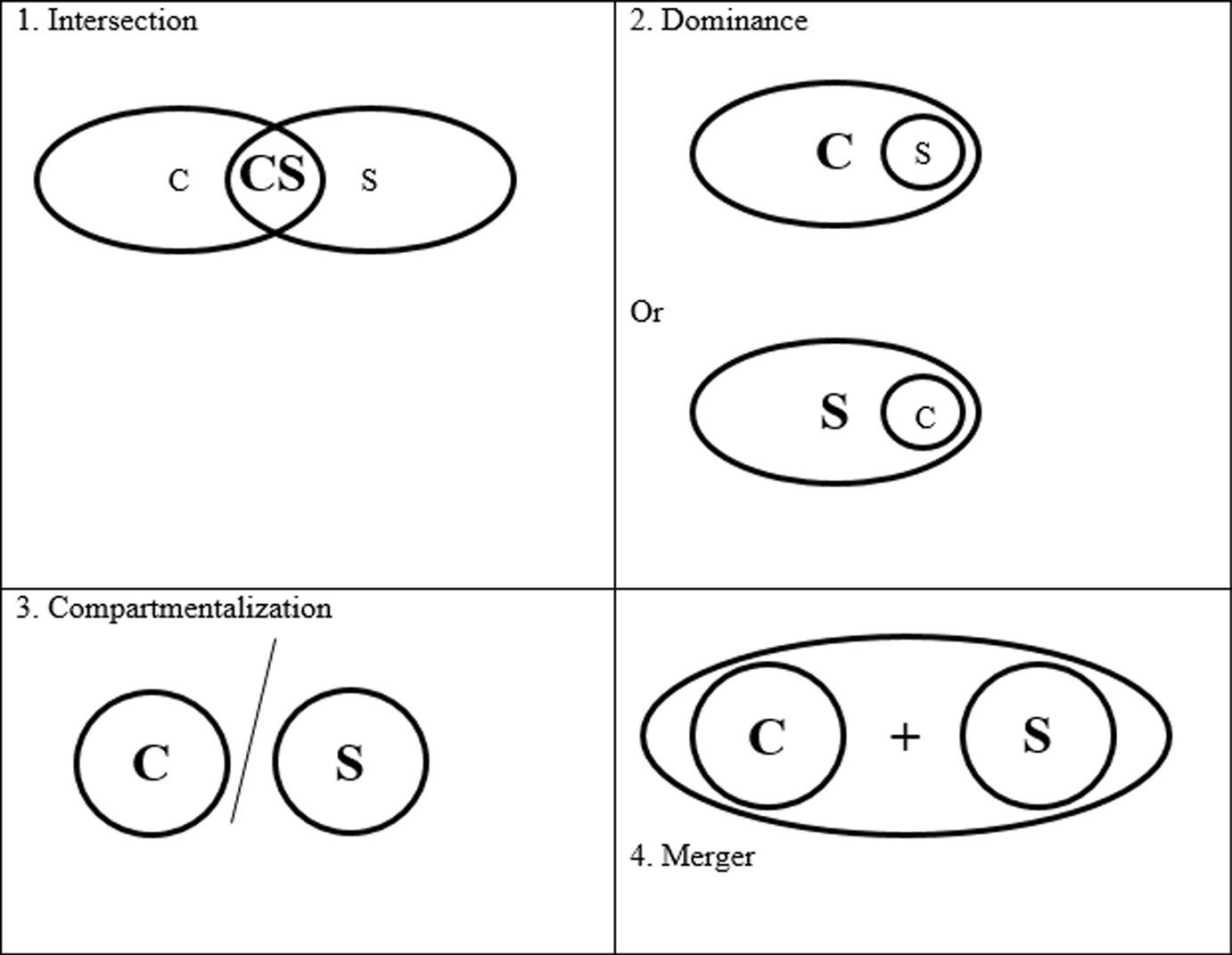
Social identity formation: potential identity forms in light of multiple group identities. (Adapted from Roccas and Brewer 2002)

Roccas and Brewer (2002) developed four representations of how multiple social identities, which result from belonging to different groups, can be combined. Each in-group representation has consequences for how a person includes or excludes others (see Fig. 1). In this study, we apply their model to CSs. First, combining multiple identities can be done by CSs by making one identity dominant over other identities. A CS with a *dominant identity* as a clinician would describe her/himself as primarily a clinician, who has scientific research as a particular task within that identity. In this example, other clinicians are recognised as in-group members. When the researcher identity is dominant, the perceptions are reversed. Second, defining one’s identity can be done as a singular identity at the intersection of both group memberships. A CS with an *intersected identity* would argue to be a “clinician-scientist” for whom both separate disciplines are not adequate to describe their identity (i.e. clinicians and scientists as out-group members). Third, in a *compartmentalised* identity CSs may embrace each social identity as in-group and make one’s identity situation- or context-specific. At the care facility, in interaction with patients, a CS may perceive his/her identity as a clinician dominant. In contrast, during a research convention, s/he may predominantly view her/himself as a researcher. Finally, a CS with a *merged identity*—the most complex social identity structure—identifies clinicians, scientists and CSs as in-group members. As such, a merged identity encompasses multiple identities—that are not easy to converge—simultaneously. These multiple identities allow the CSs to both acknowledge and accept differences across professional worlds, while simultaneously being capable of reconciling those inconsistencies and dealing with them.

### Types of CSs’ Brokerage

**Fig. 2:**
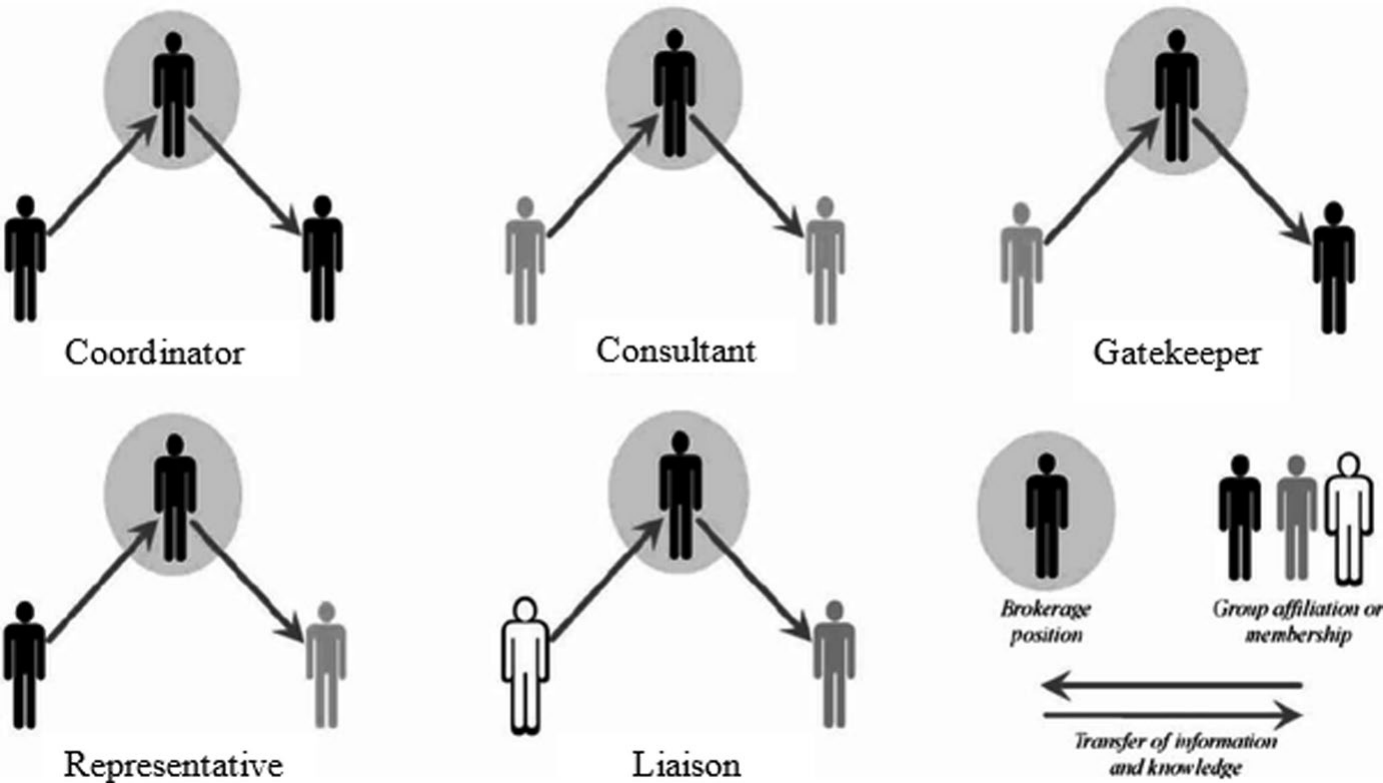
Brokerage types (Adapted from Gould and Fernandez 2006)

CSs are not only thought to act in two worlds, but they are also -often implicitly- expected to connect these worlds by brokerage. Brokerage can be thought of as a relationship or an exchange between three components in a network. Such components might be actors or sources of information. By occupying a strategic position in a network of professionals, CSs may facilitate (or hinder) exchange between traditionally distinct, separated professional worlds. As such, a theory from the network literature seemed warranted to advance our understanding of CSs’ brokerage. Gould and Fernandez (2006) have described a theory of distinct brokerage types in a network connecting people or disseminating sources of information between people who are (non-) similar to the group to which the broker belongs (see Figure 2). The conceptualisation of Gould and Fernandez might help unravel how CSs enact their brokerage. Their conceptualisation also incorporated how the broker perceives being (non)part of these groups, similar to what is considered in-group and out-group by Roccas and Brewer (2002), and therefore might be helpful to study how identity is associated with the brokerage.

The first of the five distinct brokerage types is a *liaison.* As a *liaison*, a CS may broker through connecting actors from different professional worlds. The most obvious example would be a situation in which a CS connects a clinical practitioner with a scientist. Second, as a *gatekeeper*, a CS may filter, translate, or block the exchange of information coming from one group to another group. For example, a CS may act as a gatekeeper when filtering publications and sending only the most relevant to clinical colleagues. Third, as a *representative*, a CS may broker by representing one world when interacting with the other. A CS may represent clinical practice in an academic setting by asking critical questions about the feasibility or relevance of research for practice. Fourth, as a *consultant*, a CS may facilitate exchange between professionals within one world to whom the CS her/himself does not belong, for example when connecting policymakers that share an interest in clinical practice. Fifth, as a *coordinator*, a CS exchanges knowledge primarily within the same social group of clinicians or researchers. As we focused on knowledge exchange between multiple diverse groups (specifically clinical practice and research), the single-group perspective of a coordinator and the distinct-group perspective of the consultant both lay beyond the scope of this study.

Building on the theory on social identity and brokerage types, we explored how 17 experienced, practising CSs perceive their professional identity, what diverse brokerage types they belong to, and how these types are associated with their identity perceptions.

## Method

Given the explorative nature of the study, a qualitative research design was used. The standards for reporting qualitative research (SRQR) were applied (O’Brien et al. 2014). A total of 22 participants were approached, of which 17 managed to find time to participate in this study. Research participants were selected using purposeful sampling as they were asked to join because of their position as a practising CS in the Netherlands. Two types of clinicians were involved in this study, both working in primary care settings: general practitioners (11 participants, 65%) and geriatric physicians (6 participants, 35%). Five of the CSs were female (12 male, 71%). Their age ranged between 36 and 67 years.

The participants were informed about the study according to the guidelines of the ethical review board of the NVMO (Dutch Association for Medical Education). When participants agreed, they signed a declaration of consent. The data were collected through semi-structured, in-depth, face-to-face interviews of 45–75 minutes. 14 participants were interviewed in their (clinical or research) workplace; three participants were interviewed at their homes between June and August 2017. During the interviews, additional data were collected as this research is part of a larger research project (Bartelink et al. 2019).

The interview guide was designed by the multidisciplinary research team and explored participants’ social identity and brokerage. The participants were asked to provide examples in which they, as a scientist, discussed research with a practitioner. Also, they were asked to give examples in which they, as a practitioner, discussed their (experience in) clinical practice with a researcher. Furthermore, they described examples in which they connected clinicians, scientists, and/or CSs. The participants were asked to reflect on their role and responsibility as a CS in such situations. A pilot study was performed to test the interview guide. In the pilot study, authors DS and YB were interviewers; RD was—as an author and experienced CSs—interviewee. Such preparation within the team of authors helped to include the view of the participants in the study in a solid way (Taylor-Powell and Renner 2003). Final interviews were conducted by a single researcher (DS), audio-taped, and transcribed verbatim. Reflective field notes were made during and after the interviews.

The interviews were conducted, transcribed, anonymised and subsequently analysed, using NVivo 12. Building on the conceptual frameworks of social identity and types of brokerage, theory-driven coding was used. Based on the theory, an initial template was developed. During the coding process, (sub)codes were added and refined if needed—in line with the process of *template analysis* (King 2012). For instance, within the ‘representative’ type of brokerage, different forms of fulfilling that type were recognised. Those nuances within brokerage types were coded based on the data. All transcripts were coded by two researchers separately (EG, YB): they both coded descriptions of identity and brokerage. Findings were discussed in several rounds of discussion and agreed upon among the research team to ensure reliability among coders for the coding procedure for all respondents. After final coding, cross-case analysis was conducted. In order to explore the association between identity perceptions and brokerage types, a holistic assessment of the most relevant identity perceptions was made, based on quantitative overviews generated with NVivo (percentage of coverage and comparisons between respondents), and matrix queries were carried out with coding of expressions that reflected brokerage types. Eventually, after selecting relevant example quotes, these were translated into English by a native speaker. All respondents are referred to as ‘he/she’ and identified with numbers in the results section to safeguard the anonymity. Because researchers from different backgrounds (academic medicine and educational science) participated in data analysis, we built on the strength of including diverse perspectives (Tiainen and Koivunen 2006).

## Results

The results section consists of three parts. First, we elaborate on how CSs perceive their social identity. Second, we describe their brokerage types and third, we explore how these might be associated with their identity perceptions.

### CSs’ Social Identity Perception

All four identity structures of Roccas and Brewer (2002) were encountered in the interview data. Some CSs perceived themselves as predominantly a researcher or a clinician, referred to in this study as CSs with a dominant identity. They expressed, explicitly or more implicitly, one of the identities over the other and were often very well capable of motivating their preference. For instance, CS-4 perceived her/himself as primarily a researcher: “*And besides that, I like being able to deepen my knowledge of my field. If you’re just at work in the practice, stuck in the usual routine, you have very little time to grab a book or read a guideline carefully. Or read through an article*”. This participant is not only getting energy out of doing research but also misses this stimulant in her/his clinical work. In contrast, CS-17 perceives her/himself more a clinician: “*My role is mainly to ensure adequate input from clinical practice and that the research can be done well in clinical practice, so to say. That it’s adjusted in such a way that it fits well and—the other way around—that when we work on articles, we report what the consequences are well for clinical practitioners*”. This participant considers her/himself an “*investigative practitioner*” and emphasises the importance of an investigative stance for the clinical practitioner.

Other CSs cope with their multiple social identities in three ways: intersectioning, compartmentalising or merging. Each of these three ways are characterised by whom they consider as in- or out-group. Some expressed they felt connected with other social identities or negatively expressed themselves about others: for instance, clinicians who were described as dull and uninspiring or researchers as ‘ivory tower’ theorists without including any relevance for clinical practice in their research projects. An *intersected* identity was described by CS-9 who, when asked whether (s)he felt more like a researcher or a clinician, said: “*Neither. I feel like someone standing on a bridge*”. Later in the interview, (s)he added that this means: “*You can come up with better questions when you are a GP and a researcher but most of all when you’re a GP scientist*”. (S)he also explained why (s)he maintained most professional relationships with other CSs: “*Only a very few peers are doing both [research and practice]. You have a strong bond with them, which is logical. With just clinicians or just researchers, there is less of a connection*”.

We also found evidence of CSs who *compartmentalised*. These CSs seemed to strategically choose when to respond from a specific identity, given the particular context or situation, switching in-group and out-group positions. As CS-6 explained: “*So depending on what the situation calls for, you open a certain register or not. When I’m working in the practice with my colleagues, I close all the research registers and only open my practice register for a while when I’m actually interacting with my colleagues, because they’re not interested in the other [registers]. But in my contact with patients, I do think I open that register because then you also use your scientific knowledge and insights*”. This CS explicitly explained to switch identities depending on the situation. CS-1 also spoke of ‘switching hats’, but doing so within the same context. She/he mentioned: “*[They’re] always joking like, ‘There (s)he goes again, wearing all her/his different hats’. Because it’s how I work, that’s how I talk to them. I say: ‘Now I’m putting on my GP’s hat’, or my research coordinator’s hat… or the researcher’s hat. And then I talk to them from those different perspectives*”. In this example, not only the CS her/himself, but also her/his colleagues recognise and acknowledge her/his multiple identity. In such an identity, the clinician, scientist and CS identities exist parallel to each other. All identities are of equal importance to CS-1 and (s)he is capable of acknowledging the differences between the distinct worlds, while, at the same time, reconciling them. Such professionals with a *merged identity* feel that they belong to all groups, all of the time. We had difficulties in identifying a merged identity. Some respondents did *not* express themselves negatively about researchers or clinicians. Respondents who speak more about ‘switching hats’ differ from those who found it impossible to split these perceptions (“I feel both, at the same time, I cannot separate them”). However, in the cross-case comparisons, these findings were not consistently visible in specific respondents (who used specific out-group expressions, for example).

### CSs’ Brokerage Types

Of the five potential brokerage types described by Gould and Fernandez (2006), we used three in our analysis: representative, liaison and gatekeeper. In our data, all three types emerged, but these types were more diverse. In the original framework, belonging to the same group or not as the people with whom the broker interacts is essential.

Brokerage of the type of acting as liaison was by several CSs described as being at the core of being a CS. For instance, CS-9 summarised this as “*Building bridges, connecting, and definitely spreading love for the field of clinical practice, love for knowledge acquisition, knowledge sharing, and learning from one another*”. Or, as CS-1 formulated it: “*Although I have good ideas, my job is connecting people. I like to surround myself with top scientists, who know a lot about a certain topic, and then throw clinicians in the mix, and then myself. And then we’re going to connect*”. Besides, CS-11 provided a concrete example of how the brokerage type of liaison can be fulfilled “*[…], he’s a good friend of mine and he […] was interested in heart failure. And he liked what I’d done [a PhD]. So I connected him to the centre, with people active in that area. And, in the end, that led to a PhD project*”.

CSs utilise the brokerage type of gatekeeper when they take control of the flow of resources between research and practice. For instance, CSs refer to gatekeeping from the position of a researcher, for example, by granting access to literature, as illustrated by CS-3: “*And sometimes they ask me to look up something on PubMed, for example, because they don’t have access themselves. Or they do have access, but can’t read the whole article. […] Then I have more opportunities to… and the tools and skills to… to answer questions from practice. That’s what I use academia for*”. This is a typical gatekeeper position, because the CS, as a researcher, can decide whom (s)he may grant or deny access. On other occasions, the gatekeeper type had a less visible character. CSs take part often in guideline development committees or act as reviewers for journals that are being read by clinicians; as such, CSs influence what research findings are communicated with a higher priority toward clinicians.

In the brokerage type of being a representative, we singled out distinct variations. Some CSs aim to represent clinical practice in research and vice versa; research in clinical practice. For instance, CS-7 explained that: “*…for instance, at meetings of clinicians, yes, I often put many things forward because I’ve got a very broad scope. Because of my big network and because I am sometimes ahead of things and because I think maybe we should have a look at this or I heard about this recently or maybe we should read that. […] So that’s how you just put forward things that are mainly fuelled by the university function*”. Here, the CS mainly seems to represent research in clinical practice by informing clinicians of relevant research. Other CSs seem to fulfil the type of representative more firmly, for example through posing critical questions. This is illustrated by CS-15, who represents clinical practice in research: “*… but what do we have? Or going back to the start, what’s the purpose, what’s the clinical relevance? How does it benefit the patient or, the potential practitioner in the hospital?*”

### Associations of Social Identities and Brokerage Types

In our study, social identity theory was chosen as a theoretical lens because social identity affects how a person acts in and between distinct professional worlds and thereby may help us understand whether or not a person acts according to a specific brokerage type. Brokerage was considered relevant as connecting the different worlds of clinical practice, and research is vital in the role of CSs—but the complexity of this role is often underestimated. We explored which brokerage types seemed more prevalent in respondents that had specific identity perceptions. We compared four respondents with dominant researcher identity perceptions, four respondents with dominant clinician identity perceptions, and eight respondents with identity perceptions that were intersected, compartmentalised or merged (see above, here called non-dominant).

CSs with a dominant *researcher* identity are more inclined toward gatekeeping and less toward activities that belong to a liaison or a representative brokerage type. CSs with a non-dominant identity appear to be more involved in activities of a liaison brokerage type compared to the CSs with dominant identities. All of these non-dominant identity CSs alternate within their representative brokerage type. On the one hand, they keep a close eye on the clinical relevance of the studies that researchers design. On the other hand, they make clinicians aware of the importance of (being involved in) research in the domain of general practice and elderly care medicine. *“Yes, I see it as a responsibility to conduct good research that is clinically relevant too. So, I try to keep the connections going as well as possible. So, it’s not just derived from the research setting [and turned into] something academic… eh … theoretical discussions that don’t have much to do with practice. […] But in practice I also want to keep away from the ‘well now, in my experience it’s this or that’ or ‘I’ve heard that this or that works’. That’s expert opinion and hearsay, so that you… well… there’s a big world of publications [on] evidence-based medicine. And you’ll surely find clinically relevant answers in there”. (CS-3)*

## Discussion

In this study, identity was investigated from a social identity perspective and how this shaped the types of brokerage. We investigated how CSs perceive and act upon their in-and-between position between research and practice. The CSs from this study seem to be driven to brokerage because of their eagerness and passion towards the development and use of evidence for clinical practice. This is similar to what was found among early-career CSs (Kluijtmans et al. 2017). CSs differ in perceptions of their identity and also in the activities that are critical for different brokerage types. Taken together, the involvement of CSs in brokerage varies in types and strength, related to their dominant- or non-dominant social identity.

Earlier literature (Kluijtmans et al. 2017; Rosenblum et al. 2016) has pointed towards the importance of developing an integrated CSs’ social identity. Our empirical data support this claim but provide an indication that such an integrated identity is not a merged identity. Nevertheless, it was somewhat difficult to distinguish, using their in-group or out-group perceptions, clearly between an intersected and a merged identity. Our results suggest, however, that compartmentalising CSs adapt their perspective to the environment; they take the clinician perspective strictly in clinical practice and the scientist perspective in an academic world. Other CSs tend to occupy a contrasting perspective, depending on the environment in which they find themselves; they take the clinician perspective in an academic world and a scientist perspective in clinical practice. This switching has been described in the social identity literature, starting with the work of Roccas and Brewer (2002). We contribute to the literature about how CSs connect different worlds by identifying this non-dominant identity.

Our results provide insights that CSs employ behaviour that fits into a variety of broker types while in the network literature these types of brokerage have often been described as (a more strict) position, primarily related to the structure of networks (Gould and Fernandez 2006). Within these types of brokerage, on a more detailed level, we found variety in how the brokerage was enacted—for instance, as a representative the CSs pose more or less critical questions toward research or clinical practice. These nuanced insights add empirical results to the network literature. In their review, Obstfeld et al. (2014) introduced brokerage as a social process within networks. Similarly, our results help in understanding brokerage as a dynamic process, or a continuum, with brokerage types in which CSs can be more or less involved.

Besides, our study sets the ground for further research on brokerage types. In the Fernandez-Gould framework, originated in the social network literature (Gould and Fernandez 2006; Obstfeld et al. 2014), a broker is traditionally defined as an individual acting ‘in-between’ two (groups of) individuals that (not) differ from what the broker is her/himself. The framework provided to be helpful to understand CSs’ brokerage. However, the narrow definition of ‘group membership’ as described in the original literature may have to be more broadly interpreted when applied to the context of hybrid professional roles. This study suggests that psychological membership, the feeling of affiliation with multiple groups without actually perceiving to be a full member of a group, may also define CSs’ brokerage between groups.

Social identity structures are relevant in understanding how CSs perform brokerage. Differences in how CSs cope with their multiple identities, in particular, whether they see one of these as dominant or not, lead to differences in types of brokerage they utilise. With a *non-dominant identity* (merged, intersected or compartmentalised) the CSs perform activities that belong to a diversity of brokerage types: a liaison, a gatekeeper, or a representative. CSs with a *dominant identity,* clinician or researcher, perform activities that belong to just one brokerage type primarily. A CS who mainly identifies her/himself as a clinician (*dominant identity*), acts more prominently as a representative and takes a critical stance (questioning the value for daily practice) towards researchers who want to research in clinical practice. CSs with a dominant research identity are mainly involved in translating research to clinical practice, supporting the implementation of research findings and designing research that fits daily work in clinical practice (gatekeeper). This diversity of brokerage types of CSs with a non-dominant identity may be explained by the fact that they are perhaps better able to consider, incorporate and reconcile the different stakes of the different groups they represent, allowing them to balance in and between those worlds. CSs with such an identity accept differences between professional worlds, allowing them to cope with conflicting issues which helps in brokerage. Because of this versatility, development of a non-dominant identity seems to be the preferred direction for identity development of CSs.

## Practical Implications

Currently, in most clinical and academic settings, being a CS is not a formally recognised profession (Hendriks et al. 2019). This is problematic, especially when it comes to identity formation (Rosenblum et al. 2016; Weggemans et al. 2019). The results of this study emphasise the complexity of the identity of CSs, which is specific for their unique position (Kluijtmans et al. 2017; Vignola-Gagné 2014). In the light of this broker identity, the road toward recognising CSs as a profession should not primarily, as Hendriks and colleagues (2019) have mentioned, focus on skills and competencies but also consider what is needed for identity development. Education and professional development could support in creating synergy in identities, rather than the tension between the identity as a clinician and a scientist (Lemoine 2008). Also, our results indicate that CSs adjust their perspective to the environment. This adjustment would benefit from, what Hendriks and colleagues (2019) have called, a *translational arena* in which both clinical as well as academic efforts—and especially those tasks linking these two professional worlds—are acknowledged and rewarded.

For professional development, focussing on CSs who act as a mentor might be key (Lemoine 2008; Rosenblum et al. 2016; Yanos and Ziedonis 2006). Rosenblum and colleagues (2016), for instance, argue that mentorship can help CSs in building an identity that supports them to reflect on their unique position, to accept ambiguity and complexity, and to deal with it. Deliberate exploitation of the in-between position may strengthen CSs’ position and career. Research shows that people tend to evaluate role models concerning themselves: whether or not they would like to be as the role model (Ibarra 1999). The CSs can play an essential and crucial role in attracting young clinicians and researchers to choose for a dual-career as a CS. A recent study also showed that role modelling could take place informally at the workplace, for non-CS colleagues, for instance, when clinical colleagues take an example of the scientific perspective of the CS (Van Dijk et al. 2018).

### Limitations and Future Research Directions

Although the results of this study contribute to the understanding of the social identity and brokerage of CSs, we see limitations and formulate several questions that warrant further research in CSs’ multiple positions. We only collected data in a single point in time. It would be valuable to investigate the identity formation and brokerage of CSs over time, as neither identity nor brokerage are static constructs (Taylor-Powell and Renner 2003). The identity of CSs might change over time and, because of the high potential of an integrated (non-dominant) identity, future research could investigate what kind of interventions are needed to support CSs in developing such an integrated identity. CSs acting as a mentor or role model might help here.

Furthermore, we only included experienced CSs in our study. That is, we did not include CSs that decided—at a certain point in their career—to proceed as either clinical practitioner or scientist. In this context, it must be noted that by inviting practising CSs, we only included those that sustained their position—given the complexity of the dual-discipline career. Although it would be interesting to investigate how they differ in their identity and role perception from the resilient CSs, they were not part of this study. Nevertheless, as a consequence, our results might be coloured by “survivor bias”. As Win (2017) argues, fulfilling the role of CSs is very demanding, and many CSs might choose to continue their career in the one they feel most passionate about; either in clinical practice or science. However, this does not necessarily mean that these professionals do not fulfil a brokerage type anymore (Win 2017). In the longitudinal approach, advocated for in the previous section, it would be possible to map their career path and the decisions that they make along that path. To deepen and broaden our understanding—whether they decide to continue their career as a CS, clinical practitioner or scientist—of their identity and brokerage in connecting both worlds.

This study contributed to our understanding of the highly complex and valuable position of CSs by elaborating on brokerage and their social identity. *The* CS does not exist: experienced CSs differ both in how they perceive their identity as well as in how they undertake activities that characterise certain broker types. Our results suggest that CSs may have a dominant or a non-dominant identity. Some CSs, with a non-dominant compartmentalised identity, tend to fulfil brokerage more or less pronounced over contexts (i.e. clinical practise and academia) and situations (e.g. fulfilling the type of representative, liaison, etc. in different ways). Our results provide reasons to believe that CSs with a non-dominant identity can use the full potential of brokerage, as they undertake activities that characterise the three different brokerage types. Dominant identities are more limited in the diversity of their brokerage types. Future research is needed further to disentangle the differences between merged and intersected identities. A relevant difference between the two in the uptake of brokerage types is to be expected, based on the fact that these identities differ in who they consider in-group and out-group.
